# The Influence of Milk Type on the Proteolysis and Antioxidant Capacity of White-Brined Cheese Manufactured from High-Heat-Treated Milk Pretreated with Chymosin

**DOI:** 10.3390/foods8040128

**Published:** 2019-04-17

**Authors:** Miroljub Barac, Mirjana Pesic, Sladjana Zilic, Milenko Smiljanic, Ivana Sredovic Ignjatovic, Tanja Vucic, Aleksandar Kostic, Danijel Milincic

**Affiliations:** 1 University of Belgrade, Faculty of Agriculture, Nemanjina 6, 11081 Belgrade, Serbia; mpesic@agrif.bg.ac.rs (M.P.); isredovic@agrif.bg.ac.rs (I.S.I.); tvucic@agrif.bg.ac.rs (T.V.); akostic@agrif.bg.ac.rs (A.K.); danijel.milincic@agrif.bg.ac.rs (D.M.); 2 Maize Research Institute, Slobodana Bajica 1, 11081 Belgrade, Serbia; szilic@mrizp.rs; 3 Faculty of Technology, Zvornik, 75400 Karakaj, Bosnia and Herzegovina; milenkos74@gmail.com

**Keywords:** proteolysis, goat cheese, cow’s milk cheese, antioxidant capacity

## Abstract

We investigated the effect of milk type on the proteolysis and total antioxidant capacity (TEAC) of white-brined cheeses prepared from high-heat-treated (90 °C, 10 min) cow’s and goat’s milk, pretreated with chymosin at a low temperature (4 °C). The cheeses produced showed improved antioxidant characteristics and a high content of denatured whey proteins. However, these characteristics depend on the type of milk and the ripening time. Ripened cow’s milk cheese had higher values of WSN/TN (water-soluble nitrogen per total nitrogen content) and TCA-SN/TN (nitrogen soluble in 12% trichloroacetic acid per total nitrogen), but similar PTA-SN/TN (nitrogen soluble in 5% phosphotungstic acid per total nitrogen) values were observed in ripened cheeses. The antioxidant potential of a WSF (water-soluble fraction) was higher in goat’s milk cheese, but higher TEAC (total antioxidant capacity) values of WINF (water-insoluble fraction) were observed in matured cow’s milk cheese. In vitro digestion slightly improved the radical scavenging capacity of WSF, whereas digested WINF had more than twice the capacity of their undigested counterparts. The cheeses prepared in this study could be a good source of antioxidant peptides. Further investigations of their in vitro and in vivo functionality need to be conducted.

## 1. Introduction

White cheese in brine is widely consumed in southeastern European countries. A specific aspect of this type of cheese is that maturation occurs in salt brine, usually for one or two months [1]. Today in Serbia, it is commonly made from thermally-treated cow’s or sheep’s milk, and to a lesser extent from goat milk. However, in the last 15 years, due to its nutritive and health benefits, there has been an increased interest in goat milk production and its conversion into high-valuable products, such as cheese. Usually white-brined cheese production uses a mild heat treatment. However, in the past 20 years, a higher thermal treatment of milk (above 70 °C) has been recognized as a method for improving the yield and nutritive characteristics of cheeses through the formation of so-called micellar whey protein‒casein (WP‒CN) complexes and their incorporation into the gel matrix. Their composition, degree of formation, and incorporation are influenced by several factors including the type of milk, characteristics (composition, pH), pretreatment, and heating conditions (temperature and duration of heating) [1,2,3,4]. A high amount of whey protein incorporated into the gel matrix of white-brined cheese modifies its structure and essentially influences proteolysis as the major biochemical process during cheese ripening [5,6]. 

In the last 15 years, fermented and other dairy products have been recognized as a source of a wide range of bioactive proteins and peptides, including those with antioxidant activity [7]. A part of the proteins and peptides that have antioxidant activity originate from the milk itself [8], and the thermal treatment of milk [9], but mostly from cheese ripening [10]. Antioxidant peptides are mostly derived from caseins due to the action of peptidases released from both the starter and non-starter lactic acid bacteria (LAB) [11]. The level of their formation depends on several factors, such as type of milk and its characteristics, pretreatment, and heating conditions and increases during ripening [10]. Besides caseins, whey proteins and their proteolytic products also exert antioxidant activity [12]. Thus, the incorporation of whey proteins into the cheese matrix also improves the antioxidant capacity, nutritional quality, and general functionality of cheese. 

Renan et al. [13] recognized that pretreatment considerably increased the amount of micellar-bound whey proteins. It involved using the proteolytic enzyme, chymosin, at a low temperature, and subsequently heating cow’s milk at its natural pH (pH 6.7) to 90 °C for 10 min. It is known that cow’s and goat milk differ in casein composition, casein micelles diameter, hydration, and mineralization, which strongly influence the characteristics of the cheeses [14]. In addition, goat milk is more stable after cold-induced changes, including the dissociation of micellar β-casein, than cow’s milk [15]. Therefore, it can be assumed that chymosin pretreatment at a low temperature with a subsequent high-heat treatment can differently influence not only proteolysis, but also the antioxidant capacity of cow’s and goat cheeses. Thus, the aim of this work is to study the influence of milk type on the ripening and antioxidant capacity of white-brined cheese prepared according to a modified traditional procedure consisting of a chymosin cold pretreatment and subsequent heating at 90 °C for 10 min before chymosin renneting. These findings could be helpful for the production of cheeses with improved nutritional value.

## 2. Materials and Methods 

### 2.1. Cheese Making

White cheeses were prepared from fresh, raw goat and cow’s milk according to the procedure for traditional Serbian starter-free cheeses, which was modified as follows. Milk was pretreated as described by Renan et al. [13]. Briefly, fresh milk (20 L) was cooled down to 4 °C, thermostated for 1 h, and inoculated with 28 mL of fresh aqueous solution of recombinant chymosin (9 mg 100 mL^−1^, Maxiren, 150.000 IMCU/g, DSM Food Specialties BV, Delft City, Netherlands) for 3 h at the same temperature. Then, milk was heated at 90 °C for 10 min and cooled down to room temperature. After 1 h, the treated milk was heated to 35 °C (for 30 min) and 200 mg of CaCl_2_ and 90 mg of the same recombinant chymosin, per liter of milk, were added. Curd formation took place with in 40 min at 32–33 °C. A starter culture was not added. Once curdling was complete, the cheese mass was carefully transferred from cheese vats into the mold. After about 2 h of draining without pressing, the cheese curd was cut into pieces of 10 × 10 × 3 cm and dry salted with 3.0% (w/w) NaCl. The next day, the cheese was placed into plastic cans and covered with 8% (w/w) NaCl brine. The cheese ripened during the next 50 days at 13 °C. Cheese samples were taken every 10 days and kept frozen at −80 °C. For each type of milk, the cheese was prepared in duplicate.

### 2.2. Physicochemical Analyses of Milk and Cheese

The following methods were used to determine the basic composition of the milk: total protein (TN × 6.38; total nitrogen × 6.38) [16], dry matter [17], and fat according to the Gerber method [18]. The pH was measured with a pH meter (Consort, Turnhout, Belgium). The chemical composition of each cheese sample was determined using the following standard method: the total nitrogen content of cheese samples was determined according to the Kjeldahl method [16] and expressed as total protein in dry matter (TP/DM). Dry matter content (DM) was measured by the drying method at 102 ± 2 °C [19]. Fat content was measured according to the Van-Gulik method [20]. The pH of the cheese was measured using a pH meter (Consort, Turnhout, Belgium) in a slurry [21]. NaCl content was determined according to the Volhard method [22].

### 2.3. Assessment of Proteolysis

Proteolysis was monitored throughout ripening by measuring the levels of water-soluble nitrogen (WSN), as described by Kuchroo and Fox [23]: 12% trichloroacetic acid-soluble nitrogen (TCA-SN) and 5% phosphotungstic acid-soluble nitrogen (PTA-SN) using the Kjeldahl method [16] and expressed as a percentage of TN. All determinations were made in triplicate.

Besides these parameters, proteolysis was monitored by SDS-PAGE of Tris-HCl extracts of cheese proteins, water-soluble fractions (WSF), water-insoluble fractions (WINF), and pH 4.6-soluble nitrogen fractions, according to the method of Fling and Gregerson [24], as described in our previous studies [5,6,25]. Nitrogen fractions soluble at pH 4.6 are composed of low molecular weight nitrogen compounds, peptides, and proteins soluble at the isoelectric point of native caseins. 

### 2.4. Antioxidant Properties of Water-Soluble and Water-Insoluble Protein Fractions

The total antioxidant capacity (TEAC) of WSF (TEAC-WSF) and WINF (TEAC-WINF) of the investigated cheeses was measured based on the QUENCHER method by Serpen et al. [26], as previously described in detail [5,6]. The Trolox equivalent antioxidant capacity (TEAC) was expressed in mmol of Trolox per kg of DM.

### 2.5. In Vitro Simulated Digestion of Protein Fractions

Protein fractions of ripened cheeses were subjected to in vitro gastrointestinal digestion as described by Petrat-Melin et al. [27]. Briefly, the digestion consisted of a two-step static system with a simulated gastric phase using porcine pepsin (Sigma Aldrich, Munich, Germany) at pH 2.0 for 60 min, followed by a simulated duodenal phase. In the duodenal phase the pH was increased to 6.5 by the addition of 55 mM NaHCO_3_, and the digestion was carried out for 120 min with porcine pancreatin (Sigma Aldrich). Equal enzyme activities were used for both steps, corresponding to a w/w ratio of enzyme to protein of approximately 1 to 200. After the digestion, enzymes were inactivated by heat treatment at 90 °C for 5 min and immediately cooled in ice bath. Digested fractions were lyophilized and their TACs were measured according to the same QUENCHER method by Serpen et al. [26].

### 2.6. Statistical Analysis

All measurements were done in triplicate. Data were subjected to two-way analysis of variance ANOVA, with Microsoft Office Excel ver. 7.0 (Microsoft, Washington, DC, USA) and the comparison of means was done with Tukey’s test at *p* < 0.05. In addition, Pearson’s correlation was performed to appreciate and interpret interactions between variables through the linear correlation coefficient. Principal Component Analysis (PCA) [28] was performed using the Pearson correlation. Correlation and PCA analysis were done with IBM-SPSS v20 software (IBM Corp., New York, NY, USA). 

## 3. Results and Discussion

### 3.1. Compositional Analysis

Fresh raw cow’s and goat milk had a similar chemical composition. Fresh cow’s milk had a pH of 6.69 and contained 3.4 g 100 g^−1^ of fat, 3.27 g 100 g^−1^ of protein and 11.98 g 100 g^−1^ of dry matter whereas the pH, fat, protein, and DM content of goat milk were 6.68, 3.2 g 100 g^−1^, 3.0 g 100 g^−1^, and 11.81 g 100 g^−1^, respectively. The chemical composition of cheeses that ripened for 50 days is presented in Table 1.

The average values of all investigated parameters, except for pH, are in accordance with the results reported for fresh goat and cow’s milk cheeses of different varieties [29,30,31]. The pH of fresh cheeses (5.77 for goat milk cheese, 6.01 for cow’s milk cheese) prepared in this study was higher than the recommended values for one-day-old white-brined cheeses. It is known that about 24 h after coagulation, the pH of white-brined cheese should be about 5.0 [32]. In this study the pH of fresh cheeses was measured before salting. Also, this could be the result of the absence of starter cultures. Nevertheless, these values were in the range of those reported for traditional Turkish Urfa cheese [31] or traditional Iranian starter-free white cheese [33], but lower than the values reported for fresh cheese from overheated goat and cow’s milk [5,6,30]. Considering that the high-heat treatment was used, and no starter culture was added, the pH values of fresh cheeses were low. This could be explained by acidification, which occurs during the three hours of pretreatment and after heat treatment since the cooling down was very slow. In addition, during curdling, cutting, and molding the microbial population, probably derived from external contamination, increases [30], which can also partially contribute to lower pH values of fresh cheeses. Furthermore, fresh goat milk cheese had a significantly lower pH than fresh cow’s milk cheese. This might be partly attributed to the lower buffering capacity of goat milk due to its lower protein content (3.0 g 100 g^−1^) compared to that of cow’s milk (3.27 g 100 g^−1^). 

As expected, all chemical parameters were changed during the ripening time. The mean DM content of fresh experimental cheeses prepared from goat and cow’s milk was 46.36 g 100 g^−1^ and 44.88 g 100 g^−1^, respectively. After 50 days of ripening, both cheeses had an increased DM content (49.29 g 100 g^−1^, goat milk cheese; 48.26 g 100 g^−1^, cow’s milk cheese). The increase in DM values agrees with the results reported by Malattou and Pappa [29] and Pappa et al. [34] for Teleme cheese prepared from a different kind of milk. Alichanidis and Polychroniadou [35] suggested that the rate of moisture loss is high for 15–30 days after manufacturing due to: (a) salt uptake from the brine and (b) acidity development with concomitant reduction of casein hydration as the pH reaches its isoelectric point. However, the DM of goat milk cheese significantly (*p* < 0.05) increased during the initial 10 days of ripening and then was constant, whereas the DM of cow’s milk cheese was constant during the first 20 days, increased for the next 20 days, and decreased thereafter. These results indicated the different porosity of the casein matrix of experimental cheeses, caused by the different properties of casein micelles of cow’s and goat milk and, probably, the additional effect of enzymatic pretreatment combined with high-heat treatment and a relatively large amount of salt uptake during the initial 10 days of ripening. The experimental cow’s milk cheese seems to have a more compact protein matrix than the goat milk cheese. It is known that the native casein micelles of goat and cow’s milk differ markedly in composition, mean diameter, hydration, mineralization, and cold storage stability [14,15]. Overheating goat and cow’s milk differently affects these properties and the cheese matrix properties due to the increased content and different composition of micellar WP‒CN complexes [36]. The investigation of Renan et al. [13] showed that the pretreatment of cow’s milk with chymosin combined with overheating induces similar but more intensive changes of micelles than overheating alone. In addition, the fast uptake of a relatively high amount of salt may cause a shrinkage and decrease in porosity of the structure of the casein matrix [37], which is more prominent in cow’s milk cheese than in goat milk cheese, probably due to the less porous initial matrix. This agrees well with the trend of salt uptake and pH changes. The increased DM content of cow’s milk cheese begins after 20 days when the pH reaches values near the isoelectric point of casein and the salt content reaches its maximum. After 40 days of ripening, the DM content of cow’s milk cheese decreased from 51.20 to 48.26 g/100 g. This significant decrease in the DM content of cow’s milk cheese can be attributed to proteolysis, which results in the liberation of new amino and carboxylic groups that have an increased ability to hold water [33]. 

It is known that when cheese blocks are stored in brine, a dynamic reciprocal diffusion process begins as the salt moves from the medium into the cheese texture and water diffuses out through the casein matrix into the brine [33]. The amount of salt in experimental goat milk cheese increased during the first 10 days of ripening, then slightly decreased and remained almost constant. Opposite to this, due to the less porous matrix, salt diffusion in experimental cow’s milk cheese reached equilibrium after 30 days. 

Table 1 shows that during ripening, the pH of both cheeses significantly (*p* < 0.05) decreased to 4.87 (goat milk cheese) and 4.33 (cow’s milk cheese). The pH values obtained at the end of 50 days were similar to the values reported for ripened (60–90-day-old cheeses) East Mediterranean traditional white-brined cheeses prepared from raw and pasteurized cow’s milk (4.20–4.80) [35]. Despite the lower initial value of fresh cheese, the decrease in the pH value of goat milk cheese was considerably slower compared to that of cow’s milk cheese. In fact, the pH of goat milk cheese decreased significantly (*p* < 0.05) throughout the 20 days of ripening and then remained constant with no significant (*p* < 0.05) differences, whereas the pH of cow’s milk cheese decreased continually. The most intensive decrease in pH of both cheeses was observed after 10 days of ripening. Nevertheless, the observed values of 10-day-old cheeses were higher compared to the values usually reported for 10-day-old white cheeses. This can be attributed to the slower lactic bacteria growth and acidification caused by the high-heat treatment of the milk used in this study.

It is known that the pH and acidity of cheeses are related to salt content. A higher value of pH is correlated with higher NaCl content [34,37]. According to Table 1, more salt in cow’s milk cheese resulted in a lower pH. This can be explained as follows: As previously mentioned, the experimental cow’s milk cheese seems to have a more compact casein matrix than goat milk cheese. The compact matrix allows for the retention of larger amounts of lactic acid, which contributes to the higher acidity and lower pH of cow’s milk cheese, which is in agreement with the changes in DM during ripening.

### 3.2. Total Proteins, Nitrogen Fractions, and Protein Profiles 

**Figure 1:**
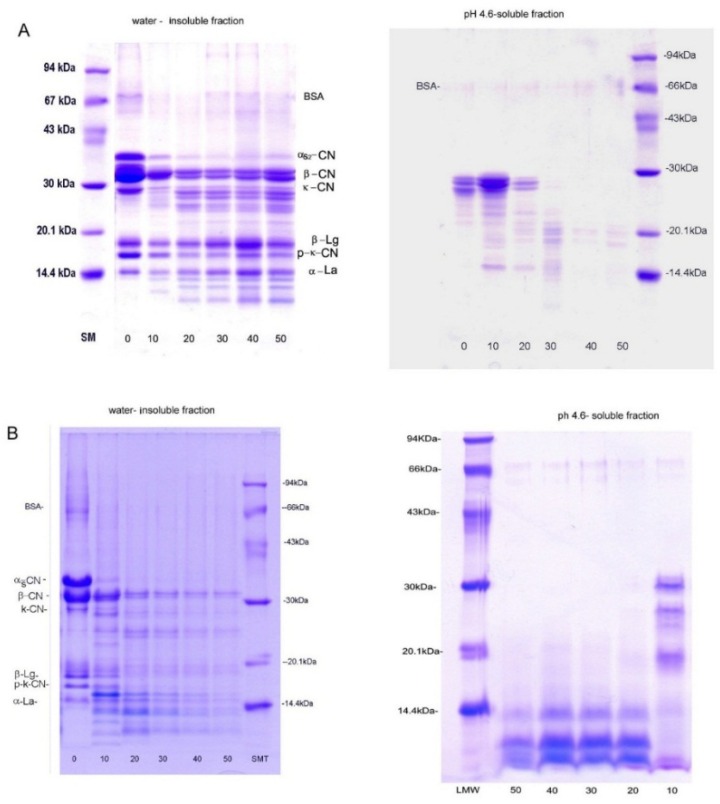
Sodium dodecyl sulfate polyacrylamide gel electrophoresis (SDS-PAGE) analysis of water-insoluble, and pH 4.6 soluble nitrogen fraction of experimental goat (**A**) and cow’s milk (**B**) cheeses *. * 0–50 days of ripening; SM: molecular weight standards.

The changes in TP/DM of experimental white-brined cheeses during ripening are shown in Table 1. The variations in the nitrogen fractions are presented in Table 2. The change of water-insoluble and pH 4.6-soluble fractions is presented in Figure 1.

The ripening of experimental cheeses is characterized by a decrease in TP/DM, which is followed by a slow increase in the WSN/TN, TCA-SN/TN, and PTA-SN contents. Table 1 shows the significantly different (*p* ˂ 0.05) TP/DM contents of fresh experimental cheeses. The average TP/DM content of fresh goat and cow’s milk cheese was 37.80 g/100 g and 40.03 g 100 g^−1^, respectively. As the protein content of fresh milk was almost similar (3.27 g 100 g^−1^, cow’s milk; 3.20 g 100 g^−1^, goat milk), the lower TP/DM content in fresh goat milk cheese could point to a slightly higher loss of protein during milk processing. Due to the diffusion of low molecular nitrogen into brine [38], throughout the 50 days of ripening TP/DM was reduced by 6.14% (goat milk cheese) and 9.04% (cow’s milk cheese). The reduction was, in general, much lower than that reported for white-brined cheese from pasteurized cow’s milk [37] and those prepared from overheated milk [5,6]. This could be attributed to the processing of milk prior to coagulation, namely, partial hydrolysis with a small amount of chymosin at a low temperature (4 °C), followed by high-heat treatment. At a low temperature, due to the reduction in hydrophobic interactions, the dissociation of β-casein, and, to a lesser extent, the dissociation of other individual caseins from the micellar structure occur [15,39]. Once in the soluble phase, β-casein can be hydrolyzed by plasmin, NSLAB proteolytic enzymes, and, to a lesser extent, chymosin. During heating, β-caseins and/or their hydrophobic fragments associate with micelles, mostly on their surface [39]. In addition, it is known that chymosin hydrolyzes k-casein into p-k-casein, even at 0 °C [15]. Renan et al. [13] demonstrated that, during the heating of milk at 90 °C for 10 min, p-k-casein interacts more readily with denatured serum proteins than k-casein. Considering all these phenomena, the increased size of casein micelles and altered cheese matrixes with a high content of whey proteins could be expected. The high intensity of bands of whey proteins detected on SDS-PAGE electropherograms of insoluble fraction of fresh cheeses (Figure 1) confirmed that they were tightly incorporated into the cheese gel matrix. 

Bearing in mind that: (1) no starter culture was added, (2) severe thermal treatment significantly reduces non-starter lactic bacteria (NSLAB), (3) the presence of β-casein and its fragments and serum protein/p-k-casein complexes on the micelle surfaces interferes with chymosin site accessibility on k-casein [38], (4) native and denatured whey proteins are resistant to proteolysis [15], and (5) β-Lg can inhibit plasmin and residual chymosin, it can be expected that the proteolysis in experimental cheeses is slower than in cheeses prepared from raw or pasteurized milk. However, the observed values of WSN/TN, TCA-SN/SN, and PTA-SN/SN are similar to the values for 60-day-old Teleme cheese prepared from cow’s and goat milk reported by Anifantakis and Moatsou [32] and Pappa et al. [34]. Nevertheless, the observed values of experimental cheeses ripened for up to 30 days were lower than reported by Mallatou et al. [40] for Teleme goat milk cheeses. The disagreement may be attributed to the high-heat treatment of milk used in this study and the absence of a starter culture. The low level of soluble nitrogen fractions observed after 30 days of ripening can be attributed to slower proteolysis, but also to the retention of whey proteins in the cheese matrix. As evident from Figure 1, whey proteins remained in water-insoluble fractions after 50 days of ripening. Table 2 shows significant (*p* < 0.05) differences between WSN/TN and TCA-SN/TN parameters during the ripening of cow’s and goat milk experimental cheeses. These parameters in goat milk cheese increased noticeably slower than in cow’s milk cheese; 50-day-ripened goat cheese had 2.46- and 5.04-fold higher values of WSN/TN and TCN/TN than fresh cheese, whereas these parameters in cow’s milk cheese increased by 8.51- and 8.01-fold. The slower increase of soluble nitrogen fractions in goat than in cow’s milk cheeses has been reported by Öner and Saridağ [41]. Michaelidou et al. [42] and Pappa et al. [34] showed that these parameters are not always reliable for the assessment of proteolysis for brine-ripened cheeses. These authors have shown that proteolysis in Teleme cheeses prepared from cow’s milk was more intensive than in those prepared from goat milk, although the values of soluble nitrogen did not show this. Higher WSN/TN and TCA-SN/TN values can indicate more intensive proteolysis in cow’s milk cheese than in goat milk cheese, but also the slower migration of soluble nitrogen compounds into brine, probably due to the lower porosity of the casein matrix and the different nature of the peptides formed during ripening. These observations were confirmed by electrophoretic analysis. As is evident from Figure 1A and 1B, the SDS-PAGE profiles of pH 4.6-soluble fractions of cheeses were significantly different. The largest part of pH 4.6-soluble fractions of ripened goat milk cheeses was composed of peptides with molecular weight in the range of 15–29 kDa whereas low MW peptides (<14 kDa) dominated the SDS-electropherograms of ripened cow’s milk cheeses. According to Ardo and Polychroniadou [21], these low molecular weight peptides (600–15,000 Da) are the major compounds of TCA-soluble nitrogenous substances, which confirms the more intensive proteolysis of cow’s milk cheese as well as the lower porosity of the casein matrix. 

The observed differences in WSN/TN and TCA-SN/TN content between experimental cheeses can also be attributed to the different casein compositions of cow’s and goat milk and their different susceptibility to residual chymosin and non-starter lactic bacteria. It was shown [34,35,40] that α_s_-caseins, which are the dominant caseins in cow’s milk, are more susceptible to proteolysis than β-caseins, which dominated in goat milk. Severe heat treatment additionally increases the rate of hydrolysis of α_s_-caseins and slightly slows down β-casein degradation [43,44], making these differences more pronounced. 

As is evident from Table 2, experimental cheeses are characterized by a similar slow increase of PTA/SN values due to the slow growth and activity of residual NSLAB. PTA-soluble nitrogen content in 50-day-ripened experimental cheeses was similar (1.35% for goat milk cheese and 1.29% for cow’s milk cheese). This is in good agreement with the results of Mallatou et al. [40]. These authors showed that the type of milk did not result in any significant differences in the PTA levels of Teleme cheeses. 

### 3.3. The Change of Antioxidant Properties during Proteolysis

**Figure 2:**
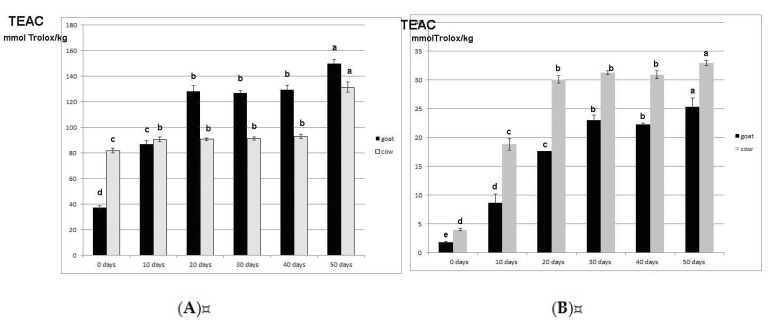
The change of total antioxidant capacity (TEAC) of water-soluble (**A**) and water-insoluble protein fractions (**B**) during the proteolysis of white-brined cheeses prepared from enzymatically pretreated and overheated goat and cow’s milk. Values with the same letters within the same graph are not statistically significant at *p* < 0.05.

In the previous section it was shown that enzymatic pretreatment combined with heat treatment differently affected the profiles of experimental goat and cow’s milk cheeses. It could thus be assumed that the experimental cheeses had different antioxidant properties. So, the change of TEAC-WSF and TEAC-WINF during the ripening of both types of cheeses was examined and the results are presented in Figure 2.

Protein fractions of investigated cheeses had significantly (*p* < 0.05) different TEAC. Furthermore, two-way ANOVA showed that milk type and ripening time both significantly affected the TEAC of WSF and WINF. In general, the WSF of ripened goat cheese had higher, whereas the WINF had a lower TEAC than their counterparts in cow’s milk. However, both protein fractions of fresh goat milk cheese had less than half the average values of TEAC than those of fresh cow’s milk cheese. In previous works of Barac et al. [5,6], it was shown that both the quantity and the properties of low molecular weight nitrogen products affected the TEAC of the WSF fraction. Since fresh cow’s milk cheese had a lower WSN content than fresh goat milk cheese, the higher TEAC of WSFs of fresh cow’s milk cheese could be attributed to their nature. Several studies [45,46,47] showed that peptides that possess free radical-scavenging activities and the ability to inhibit enzymatic and non-enzymatic lipid peroxidation were mostly derived from α_s_-caseins. The higher content of α_s_-CN in fresh cow’s milk cheese could be the key factor that accounts for the differences between the WINFs of fresh cow’s milk and goat cheeses.

Proteolysis induced an increase in the TEAC of both fractions of cow’s and goat milk cheeses but to a different extent and with different trends. The TEAC-WSF of goat cheese intensively increased during the first 20 days; after that period of ripening, the TEAC-WSF of goat milk cheese increased by more than three times and became 29.25% higher than the TEAC-WSF of 20-day-ripened cow’s milk cheese (Figure 2A). The next 20 days of ripening had no significant (*p* < 0.05) influence on the TEAC-WSF. After that period the TEAC-WSF increased and reached a maximum of 149.97 mmol Trolox kg^−1^. As opposed to goat milk cheese, the TEAC-WSF of cow’s milk cheese increased slightly (by 13.58%) during the initial 10 days of ripening, than became almost constant during the next 30 days and finally increased to 131.43 mmol Trolox kg^−1^ (50-day-ripened cheese). The differences observed between the TAC of water-soluble protein fractions of ripened goat milk and cow’s milk cheese could be attributed to a different profile of proteolysis. It is known that extensive proteolysis results in a decrease in antioxidant activity [48,49], which is in agreement with our experimental results. As is evident from Table 1 and Figure 2, the increase in WSN and TCA-SN in cow’s milk cheese during ripening was more intensive than in goat cheese. Furthermore, opposite to goat milk cheese, the water-soluble fraction of ripened cow’s milk cheese consisted mostly of peptides with a molecular weight (MW) lower than 14 kDa. These peptides, according to Pritchard et al. [50], have lower antioxidant activity. 

Ripening also improved the TEAC-WINF of both experimental cheeses. In general, the most intensive increase in the TEAC-WINF of both cheeses was detected during the first 30 days; the TEAC-WINF of 30-day-ripened cheeses were 12.87 (goat cheese) and 7.70 (cow’s milk cheese) times higher than the TEAC-WINF of fresh cheeses. Further ripening induced a slower increase in TEAC-WINF and the average TAC-WINF of 50-day-ripened goat and cow’s milk cheese was 25.53 and 32.97 mmol Trolox kg^−1^, respectively. The increase in TEAC-WINF could be attributed to proteolytic changes that made the cheese gel network more able to scavenge free radicals.

The observed antioxidant properties of experimental cheeses are due to their possible changes during digestion. In order to better understand their potential as antioxidants, both fractions of 50-day-ripened cheeses were digested in vitro. These results are presented in Table 3.

In vitro digestion affected the antioxidant properties of ripened experimental cheeses differently: digestion slightly improved TEACs (approximately up to 10%), probably due to further degradation of the low molecular weight peptides registered in Figure 1A,B, whereas TEAC-WINFs dramatically increased during digestion; the TEACs of digested WINFs were 2.30–2.53 times higher than those of undigested WIN fractions.

### 3.4. Principal Component Analysis

**Figure 3:**
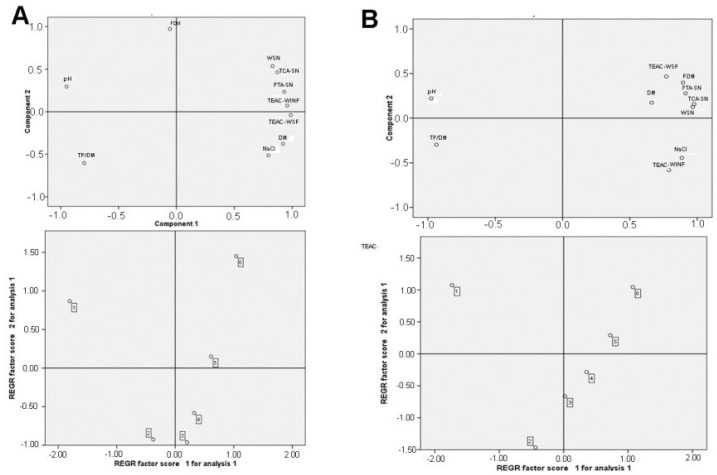
Principal component analysis (PCA) of experimental white-brined goat (**A**) and cow’s milk cheeses (**B**); PC1 × PC2, of parameters (a) and samples (b), as given in Table 1 and Table 2. Sample 1—fresh cheese; samples 2‒6—cheeses ripened for 10, 20, 30, 40, and 50 days, respectively. TCA-SN: trichloroacetic acid-soluble nitrogen; TP/DM: total protein in dry matter; WSN: water-soluble nitrogen per total protein, PTA-SN: nitrogen soluble in a 5% phosphotungistic acid per total nitrogen, TCA: nitrogen soluble in a 12% trichloracetic acid per total protein, TP: total protein in DM, FDM: fat in dry matter, TEAC-WSN: total antioxidant capacity of water-soluble fraction, TEAC-WINF: total antioxidant capacity of water-insoluble fraction.

The principal component analysis (PCA) was performed with the data gathered from physicochemical analysis of both types of cheese samples (Figure 3). For goat milk cheese, two main components (PC1 and PC2) accounted for 96.57% of the data variance. The first and second principal components explained 75.72% and 20.85% of the variance in the data, respectively. For cow’s milk cheese, similar results were obtained. (PC1 and PC2 accounted for 91.38%, PC1 79.19% and PC2 12.13%). The PC1 of both cheeses was positively associated with WSN, TCA-SN/TN, and PTA-SN/TN, which reflects the degree of cheese proteolysis, as well as with the TEAC of both fractions. Also, the PC1 of both cheeses is negatively associated with the TP/DM content and pH. In the case of goat cheese, based on close grouping, it was evident that WSN, TCA-SN/TN, PTA-SN/TN, TEAC-WSF, and TEAC-WINF had associations with each other. A similar association was observed between the parameters of proteolysis of cow’s milk cheese and TEAC-WSF. Thus, it could be predicted that the parameters of proteolysis will positively influence the TEAC of both protein fractions of goat cheese and the antioxidant capacity of WSF of cow’s milk cheese, by exerting a positive influence on the dependent component PC1. It could also be predicted that the TP/DM content and the pH will have a negative influence on the TEAC of both cheeses by exerting a negative influence on PC1. PC2 highlighted the influence of FDM (fat in dry matter) content. Through these two principal components, it is possible to separate the goat cheese samples into three groups (Figure 3A). Samples 5 and 6 (40–50-day-ripened cheeses) are located in the positive quadrant of PC1, identified by most of the characteristics related to the parameters of proteolysis. The negative quadrant of PC1 generated a second group where only sample 1 (fresh cheese) was highly influenced by the high values of parameters related to fresh cheese (pH, TP/DM). PC2 generated a third group, which consisted of samples 2, 3, and 4 (10–30-day-ripened cheeses), influenced by low FDM content. In the case of the experimental cow’s milk cheeses, the separation of samples in the score plot was similar to that for goat cheeses, and only sample 4 was associated with group 1 instead of group 3 (Figure 3B).

## 4. Conclusions

The results of this investigation clearly showed that the chymosin pretreatment of milk with subsequent overheating could be a useful method for the production of white-brined cheeses from both cow’s and goat milk. This type of cheese is characterized by a high content of denatured whey proteins, a low level of soluble nitrogen fractions, and improved antioxidant properties of both water-soluble and water-insoluble protein fractions. However, these characteristics depend on the type of milk and the ripening time. Mature cow’s milk cheese had higher values of WSN/TN and TCA-SN/TN, but the PTA-SN/TN values were similar in both cheeses (2.69 g 100 g^−1^ for goat milk cheese and 2.58 g 100 g^−1^ for cow’s milk cheese). The antioxidant potential of WSF was higher in goat milk cheese, but higher TEAC values of WINF were observed in the ripened cow’s milk cheese. Furthermore, in vitro digestion significantly improved the antioxidant capacity of WINF of both experimental cheeses and had no effect on WSFs. Based on the obtained results, it could be assumed that the white-brined cheeses prepared according to the suggested procedure may have a significant role in the maintenance of human antioxidant defense systems. Further investigations need to be conducted to confirm their health benefits. 

## Figures and Tables

**Table 1:** The effect of ripening on composition of experimental cheeses *.

Type of Milk	Time of Ripening (days)						
	Parameter	0	10	20	30	40	50
**Goat milk**	**DM (g 100 g^−1^)**	44.88 ± 0.31 ^b^	48.67 ± 0.54 ^a^	49.45 ± 0.68^a^	49.15 ± 0.15 ^a^	49.23 ± 0.36 ^a^	49.29 ± 0.20 ^a^
	**FDM (g 100 g^−1^)**	53.83 ± 0.20 ^a^	51.75 ± 0.40 ^b^	51.51 ± 0.68 ^b^	51.85 ± 1.10 ^b^	52.30 ± 0.80 ^b^	54.70 ± 0.55 ^a^
	**TP/DM (g 100 g^−1^)**	37.80 ± 0.15 ^a^	37.74 ± 0.32 ^a^	36.97 ± 0.19 ^b^	36.71 ± 0.46 ^b^	35.89 ± 0.23 ^b,c^	35.48 ± 0.18 ^c^
	**pH**	5.77 ^a^	5.01 ^b^	4.95 ^c^	4.83 ^d^	4.84 ^d^	4.84 ^d^
	**Salt (g 100 g^−1^)**	-	3.46 ^a^	3.53 ^a^	3.10 ^b^	3.11 ^b^	3.22 ^b^
**Cow’s milk**	**DM (g 100 g^−1^)**	46.36 ± 0.50 ^c^	46.91 ± 0.42 ^c^	46.42 ± 0.36 ^c^	49.44 ± 1.10 ^a^	51.20 ± 1.40 ^a^	48.26 ± 0.30 ^b^
	**FDM (g 100 g^−1^)**	50.22 ± 0.43 ^c^	50.11 ± 0.86 ^c^	54.93 ± 1.20 ^b^	55.58 ± 0.76 ^b^	56.64 ± 0.50 ^b^	60.09 ± 1.10 ^a^
	**TP/DM (g 100 g^−1^)**	40.03 ± 0.20 ^a^	39.42 ± 0.31 ^a^	38.38 ± 0.30 ^b^	38.18 ± 0.28 ^b^	36.88 ± 0.41 ^c^	36.41 ± 0.50 ^c^
	**pH**	6.01 ^a^	4.93 ^b^	4.76 ^c^	4.62 ^d^	4.47 ^e^	4.33 ^f^
	**Salt (g 100 g^−1^)**	-	3.58 ^b^	3.59 ^b^	4.11 ^a^	3.97 ^a^	3.89 ^a^

* Values with the same letters are not statistically significant (*p* > 0.05). Abbreviations are: DM: dry matter; FDM: fat in dry matter; TP/DM: total protein in dry matter.

**Table 2:** The change of ripening parameters of experimental goat and cow’s milk cheeses*.

Type of Milk	Parameter	Ripening (days)					
		0	10	20	30	40	50
**Goat cheese**	**WSN/TN (%)**	3.42 ± 0.02 ^f^	4.39 ± 0.21^e^	4.59 ± 0.08 ^d^	4.99 ± 0.11 ^c^	6.36 ± 0.05 ^b^	8.43 ± 0.09 ^a^
	**TCA-SN/TN (%)**	0.97 ± 0.04 ^e^	1.96 ± 0.09 ^d^	2.03 ± 0.10 ^d^	2.55 ± 0.13 ^c^	3.65 ± 0.21 ^b^	4.89 ± 0.11 ^a^
	**PTA-SN/TN (%)**	0.03 ^f^	0.25 ± 0.01 ^e^	0.80 ± 0.06 ^d^	0.90 ± 0.02 ^c^	1.06±0.04 ^b^	1.35 ± 0.02 ^a^
**Cow’s milk cheese**	**WSN/TN (%)**	1.57 ± 0.02 ^f^	5.69 ± 0.09 ^e^	7.86 ± 0.05 ^d^	8.21 ± 0.10 ^c^	9.23 ± 0.30 ^b^	13.36 ± 0.14 ^a^
	**TCA-SN/TN (%)**	0.75 ± 0.03 ^e^	2.50 ± 0.08^d^	3.36 ± 0.02 ^c^	4.57 ± 0.10 ^b^	5.88 ± 0.14 ^a^	6.01 ± 0.04 ^a^
	**PTA/TN (%)**	0.01 ^e^	0.34 ± 0.01 ^d^	0.63 ± 0.03 ^c^	1.10 ± 0.03 ^b^	1.15 ± 0.02 ^b^	1.29 ± 0.05 ^a^

* Values within the same parameter marked with the same letter are not statistically significant at *p* < 0.05. WSN/TN: water soluble nitrogen per total nitrogen; TCA/TN: nitrogen soluble in 12% trichloracetic acid per total nitrogen; PTA/TN: content of nitrogen soluble in 5% phosphotungstic acid per total nitrogen; TN: total nitrogen.

**Table 3:** Antioxidant capacity of protein fractions of experimental white-brined cheeses before and after in vitro digestion *.

Cheese	Antioxidant Capacity (mmol Trolox Eq/kg)			
	WSF		WINF	
	B.D.	A.D.	B.D.	A.D.
Goat	149.97 ± 5.14 ^a,A^	152.41 ± 3.18 ^a,B^	25.33 ± 1.49 ^c,D^	81.43 ± 8.14 ^b,E^
Cow	131.43 ± 5.88 ^a,B^	142.7 ± 4.21 ^b,C^	32.97 ± 1.4 ^d,C^	83.33 ± 1.33 ^c,E^

* B.D.: before in vitro digestion; A.D.: After in vitro digestion; values with the same lowercase within the same row are not statistically significant (*p* > 0.05); values with the same uppercase within the same column are not statistically significant (*p* > 0.05). WSF: water-soluble fraction, WINF: water-insoluble fraction
